# Dietary Cholesterol and the Lack of Evidence in Cardiovascular Disease

**DOI:** 10.3390/nu10060780

**Published:** 2018-06-16

**Authors:** Ghada A. Soliman

**Affiliations:** Department of Environmental, Occupational, and Geospatial Health Sciences, CUNY Graduate School of Public Health and Health Policy, The City University of New York, 55 W. 125th Street, New York, NY 10027, USA; ghada.soliman@sph.cuny.edu; Tel.: +1-646-364-9515

**Keywords:** dietary cholesterol, LDL-cholesterol, cardiovascular disease (CVD), randomized control trials (RCT), observational studies, HMG CoA reductase, LDL and HDL

## Abstract

Cardiovascular disease (CVD) is the leading cause of death in the United States. For years, dietary cholesterol was implicated in increasing blood cholesterol levels leading to the elevated risk of CVD. To date, extensive research did not show evidence to support a role of dietary cholesterol in the development of CVD. As a result, the 2015–2020 Dietary Guidelines for Americans removed the recommendations of restricting dietary cholesterol to 300 mg/day. This review summarizes the current literature regarding dietary cholesterol intake and CVD. It is worth noting that most foods that are rich in cholesterol are also high in saturated fatty acids and thus may increase the risk of CVD due to the saturated fatty acid content. The exceptions are eggs and shrimp. Considering that eggs are affordable and nutrient-dense food items, containing high-quality protein with minimal saturated fatty acids (1.56 gm/egg) and are rich in several micronutrients including vitamins and minerals, it would be worthwhile to include eggs in moderation as a part of a healthy eating pattern. This recommendation is particularly relevant when individual’s intakes of nutrients are suboptimal, or with limited income and food access, and to help ensure dietary intake of sufficient nutrients in growing children and older adults.

## 1. Introduction

Cardiovascular disease (CVD) is a leading cause of death in the US with approximately one in every four deaths occurring from heart diseases [1]. According to the CDC, 610,000 individuals die from CVD in the US [2]. The landmark of CVD is atherosclerosis, which is a chronic inflammatory condition instigated by deposition of cholesterol and fibrous tissues in the arterial walls which build up and eventually lead to narrowing and thickening or blocking of the arterial lumen. The inflammation regulates the plaque formation as well as the thrombotic complications of atherosclerosis [3]. The hypothesis that dietary cholesterol contributes to the risk of heart disease was initially suggested in 1968 and based on the research literature at the time [4,5]. Subsequently, the American Heart Association adopted a recommendation of limiting dietary cholesterol intake to 300 mg/day for healthy individuals in the United States, and with recommendations of restricting egg consumption to a maximum of three whole eggs per week [6]. However, the totality of scientific evidence and experimental data did not validate the hypothesis that dietary cholesterol increases blood cholesterol, and by extension increases the risk of CVD. Investigators have reported that increased intake of dietary cholesterol (exogenous) is associated with decreased synthesis of endogenous de novo cholesterol, possibly as a compensatory mechanism that keeps cholesterol homeostasis constant [7,8]. In fact, the 2015–2020 Dietary Guidelines for Americans removed the recommendations of setting a limit to the maximum intake of 300 mg/day cholesterol. The Guidelines still advised eating as little as possible of dietary cholesterol while maintaining a healthy eating pattern. The following review will summarize the current literature regarding dietary cholesterol, blood cholesterol, saturated fatty acids and the risk of cardiovascular disease (CVD).

## 2. Dietary Cholesterol Food Sources

Dietary cholesterol is a main steroid from animal tissues. The main food sources include egg yolk, shrimp, beef, and pork, poultry, as well as cheese and butter. According to NHANES data, the top five food sources of cholesterol in the American population (2005–2006) are eggs, and mixed egg dishes, chicken, beef, and beef mixed dishes, burgers, and regular cheese [9]. There are two main sources that contribute to and make up the liver cholesterol pool, namely dietary cholesterol (exogenous), and de novo (endogenous) cholesterol which is synthesized in the liver or extra-hepatic tissue.

The relationship between dietary cholesterol and total plasma cholesterol has been reported to be linear based on observational cohort studies [10,11]. However, the limitation of the observational studies is the presence of confounding variables that may amplify positive or negative correlations as well as the existence of selection biases [12]. Additionally, the intake of dietary cholesterol is usually associated with an increased intake of saturated fatty acids which is documented to increase LDL Cholesterol and the risk of cardiovascular disease [13]. In fact, eggs are the only dietary source of cholesterol that is low in saturated fatty acid but is also nutrient-dense, economical and affordable. The average large whole egg (50 g), contains only 1.56 g of saturated fat, 1.83 g monounsaturated fat and 0.96 g polyunsaturated fat (Table 1). Egg yolk is also rich in dietary choline (147 mg) [14], which is an essential nutrient for human liver and muscle functions [15]. Choline intake is inadequate in 9 out of 10 American Adults [16]. Additionally, choline is essential for fetal and neonatal brain development [17,18,19,20,21,22,23], and inadequate intake during these critical developmental stages is associated with negative outcomes [24,25,26]. Also, inadequate choline in pregnant women increases the risk of neural tube defects in the offspring even in the era of folate fortification of food [27,28]. As noted, each egg (50 g) contains choline (147 mg, i.e., 34% of the recommended daily Adequate Intake (AI) for adult female, and 26.5% AI for adult male) and is also rich in vitamin A (270 International Unit IU), and 80 µg Retinol Activity Equivalents (RAE) i.e., 9% RDA for adult male, 11% RDA for adult female, lutein and zeaxanthin (252 µg), folate (24 µg Dietary Folate Equivalents (DFE) i.e., 6% RDA for adult male and female), phosphorous (99 mg, i.e., 15% RDA for adult male and female), potassium (69 mg, i.e., 1% AI for adult male and female) and calcium (28 mg, i.e., 2.8% RDA for adult male and female), [16,29]. In addition to these micronutrients, the egg is also rich in high-quality animal protein (6.28 g, i.e., 11% of the recommended RDA) (Table 1).

## 3. Cholesterol Homeostasis

Cholesterol, a major sterol in animal tissues, has a significant function in the human body. Cholesterol is a structural component of cell membranes and plays an integral role in membrane fluidity. Cholesterol is also important in the synthesis of lipid rafts which are needed for protein sorting, cellular signaling, and apoptosis [30]. The characteristic structural feature of cholesterol is a fused four hydrocarbon ring referred to as a steroid nucleus, and a hydrocarbon tail consisting of eight hydrocarbon chain [31]. The cholesterol ring is the precursor of steroid hormones including estrogen, progesterone, testosterone, as well as vitamin D. As a hydrophobic molecule, cholesterol is transported in the blood via spherical macromolecules in the plasma termed lipoproteins including chylomicrons, VLDL, LDL, and HDL. The lipoproteins consist of a neutral lipid core containing cholesteryl ester and triacylglycerol surrounded by amphipathic apoproteins, phospholipids and non-esterified cholesterol. As such, the LDL particles transport cholesterol to peripheral tissues, and thus if the LDL-cholesterol is elevated, lipids can deposit in the arterial lumen leading to plaque formation, and thickening or narrowing of the blood vessel, the hallmark of atherosclerosis. On the other hand, HDL is responsible for the reverse cholesterol transport from peripheral tissues to the liver for bile acid synthesis, and steroid synthesis or for disposal of cholesterol ring via bile. As mentioned earlier, blood cholesterol is derived from two sources, exogenous dietary cholesterol and endogenous de novo synthesized cholesterol, and there is a balance and negative feedback to maintain cholesterol homeostasis. Endogenous cholesterol is synthesized by all cells and tissues, but predominantly in the liver, intestine and reproductive organs [32]. The rate-limiting and key regulatory step in endogenous cholesterol synthesis is mediated via 3-hydroxy-3-methylglutaryl CoA Reductase (HMG CoA Reductase), which reduces HMG CoA molecules to mevalonate, in the presence of NADPH as a reducing agent. Expression of HMG CoA reductase is inhibited by cholesterol as well as by statin drugs (atorvastatin, lovastatin, and Simvastatin). Thus, to maintain cholesterol balance, if dietary cholesterol absorption is increased, the endogenous synthesis is decreased [33]. The autoregulation of cholesterol synthesis encompasses control of HMG-CoA reductase by two mechanisms: (a) Feedback loop via cholesterol (which is referred to as bulk control), as well as (b) feedback loop via oxysterols, which functions to prevent accumulation of sterol intermediates and to fine tune the cholesterol regulation [34]. At the cellular level, cholesterol homeostasis is orchestrated by several regulatory transcriptional factor networks including the sterol regulatory element binding protein (SREBP), which regulates the biosynthesis and uptake of cholesterol as well as the liver X Receptor (LXR) family which regulates the excretion of excess cholesterol [35,36,37]. Another level of regulation is contributed by the farnesoid X receptor (FXR) which regulates bile acid metabolism [35,38,39,40].

## 4. Dietary Cholesterol and Cardiovascular Disease (CVD) Risk

### 4.1. Animal Models Studies

Guinea pigs are an ideal animal model to study human lipoprotein metabolism because they are LDL animals, they carry cholesterol in the LDL fraction, and they have CETP (Cholesteryl Ester Transfer Protein) similar to humans. Thus, the lipoprotein metabolism and remodeling is similar to humans. Lin et al., evaluated the cholesterol and lipoprotein metabolism in guinea pigs fed 0 dietary cholesterol (control), 0.08 (equivalent to 600 mg/day in human), 0.17 (equivalent to 1275 mg/day in human) or 0.33% dietary cholesterol (equivalent to 2475 mg/day in human) [8,41,42]. The authors reported a dose-response relationship between intake of dietary cholesterol and plasma LDL cholesterol. This effect led to decreased synthesis of endogenous cholesterol as evidenced by a reduction in HMG CoA reductase, the rate-limiting enzyme and commitment step in cholesterol synthesis, as a compensatory mechanism. The hepatic LDL receptor numbers also decreased as cholesterol concentration increased. Plasma cholesterol increased with intake of 0.17% and 0.33% cholesterol and with saturated fatty acid intake [8]. Similar to humans, guinea pigs have individualized response to dietary cholesterol, emphasizing the notion that these animals could be hypo-responders or hyper-responders to dietary cholesterol [43]. Indeed, studies in humans have demonstrated that individuals could be hypo-responders or hyper-responders to dietary cholesterol [44]. Studies conducted in 1913 in rabbits showed that dietary cholesterol in rabbits induces atherosclerosis [45]. In mice deficient in Apolipoprotein E, animals fed either a control diet (AIN-93) or a diet containing 0.2 g cholesterol or 0.2 g oxysterol, showed an increase in liver and serum levels but the dietary cholesterol did not promote atherosclerosis and did not significantly accumulate in the aorta [46].

### 4.2. Human Studies

#### 4.2.1. Observational Studies

In 1971, Kannel et al. reported that serum cholesterol was associated with increased risk of cardiovascular disease in the Framingham prospective cohort study [47]. Subsequently, risk factors for heart diseases were identified in this longitudinal study and the diet-heart disease hypothesis was established. For decades, the notion that elevated blood cholesterol is resultant from dietary intake cholesterol and saturated fatty acids were universally accepted. However, several follow-up studies showed no association between dietary cholesterol (egg consumption) and serum cholesterol, all-cause death, total coronary heart disease, or other heart disease problems such as angina pectoris or myocardial infarction [48]. Nevertheless, the recommendations of decreasing dietary cholesterol remained in effect. In 1988, Snowden reported that egg consumption was associated with all-cause mortality and coronary heart disease in females in a large cohort of California Seventh-Day Adventist adults [49]. However, Bechthold et al. conducted a meta-analysis study to investigate the relative risk between egg consumption and the risk of coronary heart disease (CHD) and stroke. There was no correlation between highest (75 g) and lowest intake of eggs (0 g) and the risk of CHD or the risk of stroke, and there was no evidence that smaller studies had an outcome reporting bias. Furthermore, in a dose-response sub-analysis with increased increments of egg intake (50 g), there was no association between egg consumption, heart disease or stroke. However, there was a positive correlation between egg intake and the risk of heart failure [50]. On the contrary, reports from the two large prospective cohort studies, namely, the Nurses’ Health Study (1980–1994) and the Health Professionals Follow-up Study (1986–1994), indicated that intake of dietary cholesterol consumed as one egg per day was not associated with increased risk of CHD in healthy men and women. Similar findings were summarized by Kritchevsky and colleagues [51,52]. In a recent study (2018), Li et al., reported that in Guangzhou Biobank Cohort Study, there were no statistical differences in the adjusted hazard ratio in all-cause mortality, mortality from CVD, ischemic heart disease or stroke and intakes of high egg consumption (7+ eggs per week) versus low egg consumption (<1 egg per week), in a meta-analysis study design that included 28,024 participants without heart disease at the time of participation [53].

Regarding patients diagnosed with diabetes, investigators of the Health Professional Follow-up Study and Nurses’ Health Study reported that in diabetic subjects, a high intake of eggs was correlated with increased risk in diabetic men [54]. Geiker et al. reviewed the literature regarding egg consumption in diabetic subjects and reported that dietary cholesterol and egg consumption was associated with increased risk of CVD [55]. However, Tran et al. did not find a correlation between egg consumption and CVD in diabetic patients [56]. In a cross-sectional study of 130,420 adult subjects aged 40–69 years in China, Shin and colleagues reported that a consumption of 7 eggs/week decreased the risk of metabolic syndrome (OR 0.77 CI, 0.70–0.84) compared with an intake of 1 egg/week [57]. Similarly, Park et al. analyzed Korea National Health and Nutrition Examination Survey (KNHANES) and found that the consumption of 4–6 eggs/week was associated with decreased risk of metabolic syndrome compared to the intake of 1 egg/month (OR 0.82, CI 0.71–0.95) [58]. However, in the US, most cohort studies reported to date suggest either a negative correlation between egg consumption in diabetic patients or no effect. For example, Djousse et al. followed a cohort of 20,703 men from the Physician Health Study and 36,295 women from the Women Health study for 20 years. The authors reported that consumption of 7 eggs/week was associated with increased risk of type 2 diabetes (Hazard Ratio 1.58, CI 1.25–2.01) in men and (1.77, CI 1.28–2.43) in women, compared to participants who consumed less than one egg per week. Additionally, the risk of heart diseases and mortality was also associated with consumption of 7 eggs/week (hazard ratio 2.01, CI: 1.26–3.20) compared to groups that consumed less than one egg per week among diabetic patients in the Physician Health Study [10]. The discrepancies of these findings could be attributable to confounding variables or differences in dietary patterns between populations. Some of the limitations of the observational studies are the inability to determine causality rather than reporting associations, possible selection bias, and the presence of confounding variables.

Based on the conflicting results, the limitations of the observational studies by confounding variables and selection bias, and a lack of causality identification; it seems that additional research methodology including meta-analysis or Mendelian Randomizations of genetically determined diabetes, metabolic syndrome, and heart disease are warranted in patients diagnosed with type 2 diabetes.

#### 4.2.2. Randomized Controlled Trial Studies

A meta-analysis study of 17 randomized control trial studies conducted from 1974 to 1999, revealed that the addition of 100 mg of dietary cholesterol increased the total/HDL cholesterol ratio [59]. On the contrary, in a randomized controlled trial, Missimer et al. [60] found that the intake of two eggs per day did not have an adverse effect on heart disease biomarkers compared to the intake of oatmeal cereal. There was an increase in both HDL and LDL cholesterol, and therefore the LDL/HDL ratio, a marker to high risk of heart disease, remained constant, and thus the net cardiovascular risk did not increase. In the agreement, a randomized controlled trial (DIABEGG) compared the intake of a high-egg diet (2 eggs/day) with a low-egg diet (less than 2 eggs/day). The results showed that there were no differences in total cholesterol, LDL cholesterol or glycemic control in overweight and obese prediabetic or patients with type 2 diabetes [61]. Furthermore, to investigate the effects of egg on endothelial functions in patients with coronary heart disease, Katz et al., conducted a randomized, single-blind, cross-over, controlled trial for 6 weeks to compare breakfast containing 2 eggs, egg beaters, or a high carbohydrate breakfast [62]. The results indicated that there were no differences between groups in regards to lipids, flow-mediated dilation, systolic and diastolic blood pressure or body weight. Similarly, in a long-term randomized control trial, van der Made and colleagues [63] compared the consumption of lutein-enriched egg yolk in a buttermilk drink with a placebo group for one year. The authors reported that total cholesterol, LDL, and HDL cholesterol, as well as the total cholesterol to HDL cholesterol ratio, were not different between the two groups. Similar findings were reported in a randomized controlled trial comparing the intake of three eggs per day to Choline Bitartrate Supplement in healthy young men [64]. The study found that HDL-Cholesterol to LDL-cholesterol was maintained, indicating that exogenous cholesterol intake downregulated Sterol Regulatory element binding protein-2, and HMG-CoA reductase expression, thereby reducing endogenous cholesterol synthesis and thus maintaining the LDL/HDL ratio constant [64]. Blesso and colleagues conducted a randomized controlled trial (*n* = 40) where participants with metabolic syndrome were randomly assigned to either 3 whole eggs/day or yolk-free egg substitutes for 12 weeks, and both groups maintained a carbohydrate-restricted diet (25–30%) [65]. The authors reported that participants who consumed whole eggs showed an improved lipid profile and decreased insulin resistance [65].

Several other randomized controlled trials indicated that egg consumption increased HDL cholesterol and decreased the risk factors associated with metabolic syndrome [66,67,68]. Along the same line, Fuller and colleagues reported that consumption of a high egg diet in pre-diabetes and patients with type 2 diabetes who had energy-restricted diets, had no adverse effect on blood glucose or glycated hemoglobin [61,69]. In a systematic review and meta-analysis, Berger et al. [70] compared the results from 19 randomized controlled trials of healthy individuals consuming dietary cholesterol (doses ranged between 501 and 1415 mg/day), or control groups (0 to 415 mg/day cholesterol). The authors reported a significant effect of dietary cholesterol on both LDL-Cholesterol as well as HDL-Cholesterol. As a result, the net LDL/HDL ratio was constant (0.17 net change) [70,71,72,73,74,75,76,77,78,79,80,81,82,83,84,85,86,87,88,89,90,91,92,93]. The increase in HDL cholesterol was pronounced with dietary cholesterol interventions doses between 650 and 900 mg/day [82,83,84,85,86,87,88,89,90,91,92,93].

## 5. Dietary Cholesterol, Saturated Fat, Trans Fatty Acids, and Cardiovascular Disease

As shown in Table 1, most foods that contain high cholesterol content are also rich in animal-based saturated fatty acids (SFA). As such, for each 100 g beef (untrimmed) that contains 99 mg cholesterol, it has 29.4 gm SFA; natural cheese, 107 mg cholesterol, and 19 gm SFA; 214 mg cholesterol, and 50 gm SFA; and chicken (meat and skin) contains 101 mg cholesterol, and has 3.8 gm SFA. The exceptions are egg and shrimp. Shrimp contains 124 mg cholesterol and 0 g SFA, and one large egg (50 g) contains 186 mg cholesterol and 1.56 g SFA (Table 1). While shrimp is arguably expensive, egg is an economical and nutrient-dense food item with high-quality protein which is convenient and affordable to low-income families and is a good source of nutrients for growing children and older adults.

A large body of literature documented the negative effects of saturated fatty acids on the development of CVD as reviewed by Yu and Hu 2018 [94]. Further, the American Heart Association reviewed the scientific evidence from prospective observational studies and randomized controlled trials and concluded that the replacement of dietary saturated fatty acids with polyunsaturated or monounsaturated fatty acids decreased the risk of CVD [95]. Trans-fatty acids are found naturally in a small amount in some meat and dairy products. However, most food consumption of trans-fatty acids is from manufactured hydrogenated unsaturated fatty acids. These partially hydrogenated trans-fatty acids that introduce at least one hydrogen bond in the trans configuration were originally developed to improve the quality and shelf life of baked goods and are used in margarine and commercial cooking. However, mounting evidence indicated that trans-fatty acids increased the risk of coronary heart disease mortality and cardiovascular disease incidence in a manner similar to saturated fatty acids [96,97,98,99,100]. As such, several European Countries introduced laws to limit the amount of trans-fatty acids in food [97]. In the United States, the FDA ruled that the content of trans fat should be included in the food label since 1990. The 2015–2020 Dietary Guidelines recommended limiting the intake of saturated fat and trans fat as part of a healthy eating pattern.

Thus, the totality of scientific evidence guided the 2010 and 2015 Dietary Guidelines for Americans and developed the recommendations to decrease intake of saturated fatty acids to less than 10% of calories and replacing it with monounsaturated and polyunsaturated fatty acids. Additionally, the American Heart Association recommends less than 7% of calories from saturated fatty acids. Fielding et al. found that an intervention with an intake of 600 mg cholesterol with a diet high in the saturated fatty acids led to increased LDL cholesterol much more than when cholesterol was administered with polyunsaturated fatty acids [75]. As noted earlier, several of the high cholesterol foods are also rich in saturated fatty acids such as beef (untrimmed and with marble), natural cheese, and butter (Table 1, and the USDA Nutrient Composition Database), and thus may increase the risk of CVD due to the saturated fatty acid content. The exceptions are shrimps (zero saturated fatty acids) and eggs (1.56 gm saturated fatty acids per large egg which accounts for 0.65% of calories).

## 6. Conclusions

The current literature does not support the notion that dietary cholesterol increases the risk of heart disease in a healthy individuals. However, there is an ample evidence that saturated fatty acids and trans-fats increase cardiovascular disease risk. The fact that dietary cholesterol is common in foods that are high in saturated fatty acids might have contributed to the hypothesis that dietary cholesterol is atherogenic. In contrast, eggs are affordable, rich in protein and micronutrients, nutrient-dense and low in saturated fatty acids. The healthy eating pattern can incorporate nutrient-dense, calorie controlled meals with balanced nutrients and a variety of colorful vegetables and fruits. The body of literature regarding dietary cholesterol and cardiovascular disease in patients diagnosed with diabetes is still inconclusive and warrants further research.

## Figures and Tables

**Table 1:** Nutrient Composition of Most Commonly Consumed Cholesterol-Containing Foods

Food Item	Unit	One Egg ^a^	Beef ^b^	Cheese ^c^	Chicken ^d^	Butter ^e^	Shrimp ^f^	Two Eggs ^g^
		per	per	per	per	per	Per	per
		50 gm	100 gm	100 gm	100 gm	100 gm	100 g	100 gm
**Nutrients**								
**Energy**	**kcal**	**72**	**674**	**393**	**215**	**714**	**62**	**143**
Protein	g	6.28	8.21	25	18.6			12.56
Total lipid (fat)	g	4.75	70.9	32.14	15.06	78.57	13.27	9.51
Carbohydrate, by difference	g	0.36	-	-	-	-	0.88	0.72
Fiber, total dietary	g		-	-	-	-	-	0
Sugars, total	g	0.18	-	-	-	-	-	0.37
**Minerals**								
Calcium, Ca	mg	28	26	714	11	-	53	56
Iron, Fe	mg	0.88	0.72	-	0.9	-	0.32	1.75
Magnesium, Mg	mg	6	5	-	20	-	-	12
Phosphorus, P	mg	99	61	-	147	-	-	198
Potassium, K	mg	69	96	-	189	-	-	138
Sodium, Na	mg	71	26	607	70	-	566	142
Zinc, Zn	mg	0.65	0.82	-	1.31	-	-	1.29
Copper, Cu	mg	0.04	0.03	-	0.048	-	-	0.072
Manganese, Mn	mg	0.01	-	-	0.019	-	-	0.028
Selenium, Se	µg	15.3	6.6	-	14.4	-	-	30.7
Fluoride, F	µg	0.6	-	-	-	-	-	1.1
**Vitamins**								
Vitamin C, total	mg	-	-	-	1.6	-	-	-
Thiamin	mg	0.02	0.03	-	0.06	-	-	0.04
Riboflavin	mg	0.23	0.04	-	0.12	-	-	0.457
Niacin	mg	0.04	1.44	-	6.801	-	-	0.075
Pantothenic acid	mg	0.77	0.16	-	0.91	-	-	1.533
Vitamin B-6	mg	0.09	0.11	-	0.35	-	-	0.17
Folate, total	µg	24	-	-	6	-	-	47
Choline, total	mg	147	-	-	59.7	-	-	293.8
Vitamin B-12	µg	0.45	0.73	-	0.31	-	-	0.89
Vitamin B-12, added	µg	-	-	-	-	-	-	-
Vitamin A, RAE	µg	80	-	-	41	-	-	160
Carotene, beta	µg	-	-	-	-	-	-	-
Carotene, alpha	µg	-	-	-	-	-	-	-
Cryptoxanthin, beta	µg	4	-	-	-	-	-	9
Vitamin A, IU	IU	270	-	-	137	2857	177	540
Lycopene	µg	-	-	-	-	-	-	
Lutein + zeaxanthin	µg	252	-	-	-	-	-	503
Vitamin E (alpha-tocopherol)	mg	0.53	-	-	0.3	-	-	1.05
Vitamin E added	mg	-	-	-	-	-	-	-
Tocopherol, beta	mg	0.01	-	-	-	-	-	0.01
Tocopherol, gamma	mg	0.25	-	-	-	-	-	0.5
Tocopherol, delta	mg	0.03	-	-	-	-	-	0.06
Vitamin D (D2 + D3)	µg	1	0.3	-	0.2	-	-	2
Vitamin D3 (cholecalciferol)	µg	1	0.3	-	-	-	-	2
Vitamin D	IU		11	14	10	-	-	82
Vitamin K (phylloquinone)	µg		3.4	-	1.5	-	-	0.3
**Lipids**								
^h^ **Fatty acids, total saturated**	**g**	**1.56**	**29.5**	**19.64**	**4.31**	**50**	**0**	**3.126**
Fatty acids, total monounsaturated	g	1.83	30.9	-	6.24	-	-	3.658
Fatty acids, total polyunsaturated	g	0.96	2.56	-	3.23	-	-	1.911
Fatty acids, trans	g	0.02		-	0.097	-	-	0.038
^i^ **Cholesterol**	**mg**	**186**	**99**	**107**	**75**	**214**	**124**	**372**
**Amino Acids**								
Tryptophan	g	0.08	0.05	-	0.207	-	-	0.167
Threonine	g	0.28	0.33	-	0.767	-	-	0.556
Isoleucine	g	0.34	0.37	-	0.924	-	-	0.671
Leucine	g	0.54	0.65	-	1.35	-	-	1.086
Lysine	g	0.46	0.69	-	1.509	-	-	0.912
Methionine	g	0.19	0.21	-	0.493	-	-	0.38
Cystine	g	0.14	0.11	-	0.249	-	-	0.272
Phenylalanine	g	0.34	0.32	-	0.721	-	-	0.68
Tyrosine	g	0.25	0.26	-	0.597	-	-	0.499
Valine	g	0.43	0.41	-	0.902	-	-	0.858
Arginine	g	0.41	0.53	-	1.169	-	-	0.82
Histidine	g	0.15	0.26	-	0.544	-	-	0.309
Alanine	g	0.37	0.5	-	1.089	-	-	0.735
Aspartic acid	g	0.66	0.75	-	1.659	-	-	1.329
Glutamic acid	g	0.84	1.23	-	2.714	-	-	1.673
Glycine	g	0.22	0.5	-	1.223	-	-	0.432
Proline	g	0.26	0.39	-	0.911	-	-	0.512
Serine	g	0.49	0.32	-	0.657	-	-	0.971
Hydroxyproline	g		0.09	-	-	-	-	-

^a^ Egg: the reference is one large eggs (50 g), 01123, egg, whole, raw, fresh. ^b^ Beef reference is retail cut beef, 13019, beef, retail cuts, separable fat, raw; ^c^ Cheese, reference is natural cheese, 45352301, natural cheese UPC: 049646936410; ^d^ chicken: the reference is 05006, chicken, broilers or fryers, meat, and skin, raw; ^e^ Butter: the reference is unsalted butter, 45118176, White rose, unsalted butter, UPC: 074807101161; ^f^ Shrimp: the reference is shrimp raw medium-UCP = 041625114505; ^g^ eggs: the reference is two large eggs (50 g each for total of 100 g), 01123, egg, whole, raw, fresh. ^h^ Saturated fat content is in bold font, and ^i^ cholesterol content is in bold. Data obtained from the USDA National Nutrient Database for Standard Reference 1 April 2018 Software v.3.9.4 2018-05-02 [14].
